# Electrospun Fibrous Scaffolds for Tissue Engineering: Viewpoints on Architecture and Fabrication

**DOI:** 10.3390/ijms19030745

**Published:** 2018-03-06

**Authors:** Indong Jun, Hyung-Seop Han, James R. Edwards, Hojeong Jeon

**Affiliations:** 1 Botnar Research Centre, Nuffield Department of Orthopaedics, Rheumatology and Musculoskeletal Sciences (NDORMS), University of Oxford, Oxford OX3 7LD, UK; indong.jun@ndorms.ox.ac.uk (I.J.); hyungseop.han@ndorms.ox.ac.uk (H.-S.H.); james.edwards@ndorms.ox.ac.uk (J.R.E.); 2 Center for Biomaterials, Korea Institute of Science & Technology (KIST), Seoul 02792, Korea; 3 Division of Bio-Medical Science & Technology, KIST School, Korea University of Science and Technology, Seoul 02792, Korea

**Keywords:** electrospinning, nanofiber scaffolds, tissue engineering, extracellular matrix-mimicking geometries

## Abstract

Electrospinning has been used for the fabrication of extracellular matrix (ECM)-mimicking fibrous scaffolds for several decades. Electrospun fibrous scaffolds provide nanoscale/microscale fibrous structures with interconnecting pores, resembling natural ECM in tissues, and showing a high potential to facilitate the formation of artificial functional tissues. In this review, we summarize the fundamental principles of electrospinning processes for generating complex fibrous scaffold geometries that are similar in structural complexity to the ECM of living tissues. Moreover, several approaches for the formation of three-dimensional fibrous scaffolds arranged in hierarchical structures for tissue engineering are also presented.

## 1. Introduction

Tissue engineering is an emerging multidisciplinary field that aims to regenerate damaged or lost tissues/organs of living organisms using a combination of cells and scaffolds [1,2]. These engineering techniques start with scaffolds, providing environments for cells/tissues to grow in an orderly manner, and become functionalized into new tissues/organs [3]. Given the importance of intercellular interactions between scaffolds and implanted surrounding cells/tissues, considerable efforts have been made to design an artificial extracellular matrix (ECM) composed of complex fibrous structures, including glycosaminoglycans, collagen, elastin, and reticular fibers [4,5,6]. These components are known to provide mechanical and biochemical support to the surrounding cells, and these effects are dependent on tissue type [7,8,9]. The biomimetic approaches taken in the field of biomaterials seek out innovation in technology from the phenomenon in nature. The relationship between architecture and function that governs normal physiology is equally instrumental in tissue regeneration. Therefore, biomaterials should be designed and engineered with the optimized structural function for the target tissue in mind.

Electrospinning has been used for the fabrication of ECM-mimicking fibrous scaffolds for several decades [10,11,12]. Electrospinning is a spinning technique that uses electrostatic forces to produce fibrous scaffolds from biocompatible polymers. A simple equipment setup makes electrospinning a versatile way to process all of the different biocompatible polymers into fibrous scaffolds. Several studies have been conducted to apply tissue engineering through controlling parameters of the electrospinning process, including electrospinning process parameters (i.e., the application of an electric field, flow rate, distance between the needle and collector, and diameter of the metallic needle) and solution parameters (i.e., concentration, viscosity, and solution conductivity) [13,14,15,16,17]. These processes can be suitable for the large-scale production of scaffolds with a controllable single fiber diameter, motivating us to attempt to manipulate the process for tissue-specific applications.

**Figure 1:**
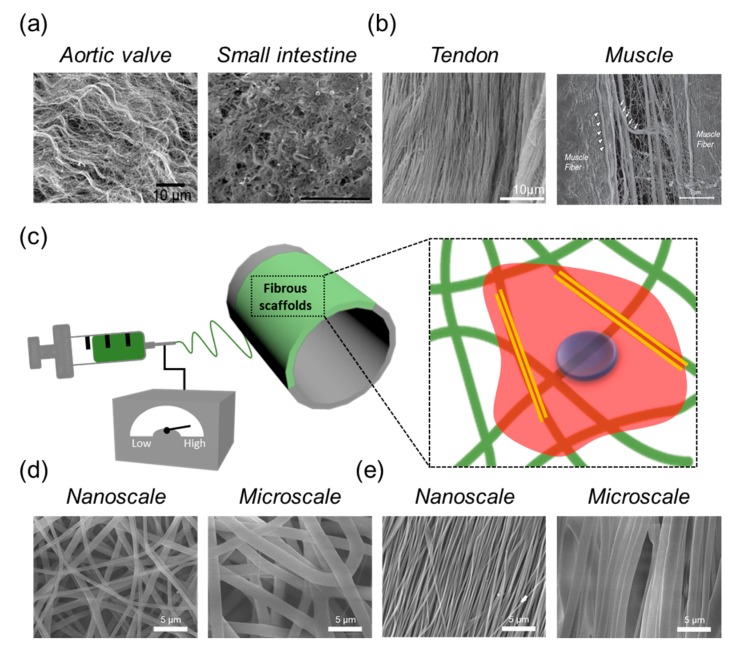
Scanning electron microscope (SEM) images of a natural extracellular matrix (ECM) in distinct types of tissues with (**a**) isotropic direction and (**b**) anisotropic direction [30,31,32,33]; (**c**) Schematic illustration of the electrospinning process; (**d**) Representative SEM images of fibrous scaffolds with a controllable fibrous scale with (**d**) randomly and (**e**) aligned fibrous deposition via electrospinning.

As with other biocompatible materials used to mimic native tissues, electrospun fibrous scaffolds provide nanoscale/microscale fibrous structures with interconnecting pores, resembling the natural ECM in tissues, and showing a high potential to facilitate the formation of artificial functional tissues (Figure 1). For example, porosity is observed in skin and bone tissues, which have a large volume fraction of interconnected pores to facilitate cell migration and the transport of nutrients during tissue regeneration [18,19]. However, conventional electrospun fibrous scaffolds are composed entirely of closed-packed fibers, which provide a superficial porous structure, and these porous structures became smaller when the fiber diameter is decreased from the microscale to the nanoscale. Such poor cell infiltration into the fibrous scaffolds results in the formation of two-dimensional (2D) surfaces rather than a three-dimensional (3D) environment, which more closely mimics the ECM. Sisson et al. demonstrated that the small pore size of electrospun fibrous scaffolds could hamper cellular migration into a fibrous scaffold, restricting tissue ingrowth [20].

To date, the scientific community has investigated a considerable number of fabricated structures of electrospun fibers and their corresponding functions for tissue engineering, including the generation of skin, bone, muscle, cartilage, and blood vessels [21,22,23,24,25,26,27,28,29]. Although these introduced fibrous scaffolds have been successfully applied to mimic native tissues, more research is needed in order to fully understand the cellular responses to sophisticated structures with different scales, dimensions, and spatial arrangements. Therefore, it is important to analyze the sophisticated structures of fibrous scaffolds, including its spatial geometry and 3D form. In this review article, we summarize the fundamental principles of electrospinning processes for generating complex fibrous scaffolds geometries that are similar in structural complexity to the ECM of living tissues. Moreover, several approaches for the formation of 3D fibrous scaffolds arranged in hierarchical structures for tissue engineering are also presented.

## 2. Fibrous Scaffolds with Hierarchical Structures

Improving our understanding of the structural features of tissues is critical for selecting appropriate materials for reconstructing damaged tissues. Within the last several decades, many studies have aimed to fabricate fibrous nanoscale/microscale structures by applying different electrospinning process parameters (Figure 1). Electrospinning works on a simple principle; a charged polymer jet is collected on a metallic collector when the applied electrostatic charge overcomes the surface tension of the polymer solution. Electrospun fibrous scaffolds are typically assembled into nonwoven networks, which are deposited in an anisotropic (random) fiber orientation. These randomly deposited fibrous scaffolds have potential applications as temporary substitutes for skin and bone tissue engineering, because they replicate the microstructure of natural tissue [21,24]. Moreover, the high surface area of electrospun fibrous scaffolds allows oxygen permeability at the wound site, making these scaffolds suitable substrates for wound dressings.

Tissues in the human body also have unique ECM structures that are found in specific tissues (i.e., the heart, nerve, and blood vessel) exhibiting anisotropic fibrous structures. From a structural viewpoint, structures that can mimic these features are believed to be necessary for precisely guiding cell growth and tissue regeneration, which lead to the recovery of their natural tissue function. For this purpose, electrospun fibrous scaffolds with various alignments have shown an outstanding ability to guide cell morphology and affect cell function when compared with other types of random fibrous scaffolds, both in vitro and in vivo. For example, Gnavi et al. demonstrated that aligned fibrous scaffolds provide contact guidance to cultured nerve cells, resulting in the alignment and elongation of cells along the contacted fiber direction [34]. Lee et al. found that aligned fibrous scaffolds could serve as ideal materials for bone tissue engineering, and that the orientation of fibers plays an important role in the guidance of new bone formation [35]. In addition, other studies have shown that myoblasts and endothelial cells are also affected by cellular morphological changes in aligned fibrous scaffolds [36,37].

**Figure 2:**
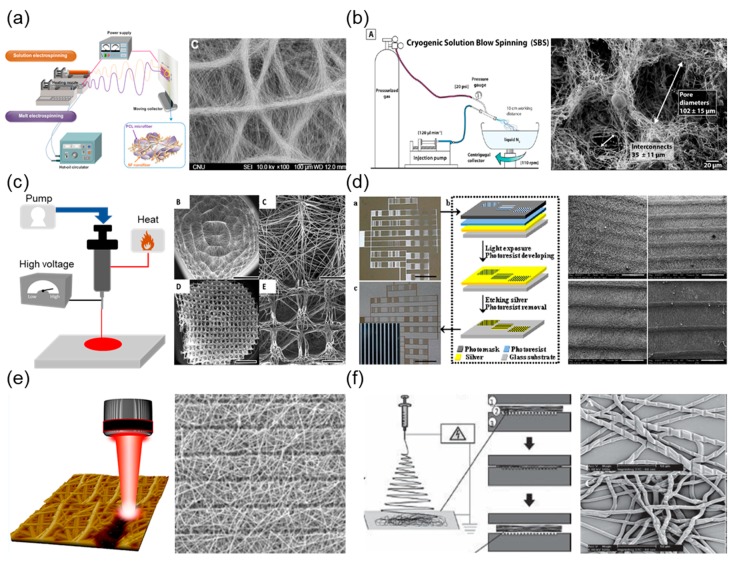
Techniques to control geometry in fibrous scaffolds and representative images. Schematic setup for **(a)** dual extrusion electrospinning [40]: (**b**) cryogenic electrospinning [41]; (**c**) melt electrospinning [42]; and (**d**) micropatterned collector-based electrospinning [43]. Schematic illustration of post-processing techniques using (**e**) laser-based ablation [44] and (**f**) nanoimprinting lithography [45].

Although numerous fibrous scaffolds with ECM-mimicking structures have been introduced to date, most studies of fibrous scaffolds have verified the effects of unitary structures, such as fiber scale, porosity, and orientation. However, most of the native tissues in humans are composed of hierarchical structures and are not unitary structures. For example, bone tissue has a hierarchical organization over various scales ranging from microscale to nanoscale-structured components [38]. Tendons are the connective tissues that bridge muscles to bone, allowing the maximal transmission of forces to produce movement at joints [39]. For these reasons, studies based on fibrous scaffolds with unitary structures may provide us with incomplete knowledge of the unitary structure, and do not consider hierarchical structures. Therefore, hierarchically patterned fibrous structures are required to design scaffolds for tissue engineering, which would allow us to better understand cell behaviors and functions on ECM-mimicking scaffolds. In this section, we discuss recent progress in the production of electrospun fibrous scaffolds with hierarchical structures via a variety of techniques (Figure 2). 

### 2.1. Geometric Control on Fibrous Scaffolds

#### 2.1.1. Dual Extrusion Electrospinning

Dual extrusion electrospinning employs two extrusion processes that function simultaneously. This technique enables the differential control of spatial geometry depending on extrusion conditions, e.g., polymeric concentrations and independent solvents. This permits nanofibrous/microfibrous structures to be combined in a single scaffold and the distribution of electrospun fibers to be controlled. Leverson et al. investigated the cellular response using hybrid scaffolds of mixed nanoscale/microscale fibers fabricated by dual extrusion electrospinning, in which one syringe creates nanoscale fibers, and the other generates microscale fibers [46]. Park et al. prepared layer-on-layer stacks that included alternating layers of random fibers and aligned fibers [47]. These proposed hybrid fibrous structures provide more stable mechanical support than single fibrous scaffolds. Furthermore, the presence of nanoscale fibers in the hybrid-scale scaffolds influences cell behaviors. For example, Kim et al. demonstrated that the osteoblast differentiation ability increased in hybrid scaffolds composed of nanoscale silk fibroin fibers and microscale poly(3-caprolactone) (PCL) fibers [40]. The authors reported that hybrid-scale scaffolds have the advantage of forming ECM-mimicking structures to support cell migration for microscale fibers, whereas nanoscale fibers were used to mimic the structure of the ECM for cell adhesion. Hybrid-scale scaffolds with multiple scales can provide synergistic effects, thereby improving the function of osteoblasts. Therefore, dual extrusion electrospinning is a suitable method for generating scaffolds with different fibrous scales and structures, which is critical for controlling the behavior of cells.

#### 2.1.2. Temperature-Assisted Electrospinning (Cryogenic/Melt)

Fibrous scaffolds can also be fabricated by temperature-assisted electrospinning, using additional temperature-controlled equipment to improve cell permeability. Cryogenic electrospinning uses ice crystals throughout the process of depositing fibrous layers in order to prevent the formation of highly compacted fibrous scaffolds during the electrospinning process. Scaffold porosity can be adjusted from 10–500 μm, depending on various controllable factors such as size and the amount of ice crystals. The ice crystals are then removed through the freeze-drying of the fibrous scaffolds, leaving large void spaces. This technique is a promising technique for developing fibrous scaffolds, permitting cell infiltration into these void spaces. Bulysheva et al. have used cryogenic electrospinning to generate fibrous scaffolds that are capable of inducing an in-growth of fibroblasts and epithelial cells into scaffolds [48]. Similarly, cryogenic solution blow spinning uses a system of concentric nozzles and pressurised gas to blow a polymer solution into a cryogenic liquid. Medeiros et al. have developed a method to generate fibrous scaffolds with controlled porous structures using a combination of thermally induced phase separation and solution blow spinning [41]. Ice microspheres create these fibrous scaffolds, forming interconnected networks with the fibers directly on the surface of the liquid nitrogen. Fibrous scaffolds made using this method have 3D scaffolds that are formed of porous fibers with interconnected macroscale pores.

Alternatively, melt electrospinning is another temperature-assisted electrospinning approach using a higher temperature. Melt electrospinning uses a polymer melt instead of a polymer solution, permitting the controlled fibrous deposition of 3D scaffolds with programmable porosity and alignment. In principle, the polymer is placed in a syringe that can be heated to a desirable temperature (~400 °C) and extruded using air pressure. This technique overcomes the disadvantages of conventional electrospinning, e.g., toxic solvents are not required [49]. This lack of solvent has implications for a wide range of biomedical materials, because the residual solvent does not need to be removed in order to use the scaffolds on cells or tissues. Zaiss et al. used melt electrospinning in order to produce 3D fibrous scaffolds in which the average pore size and fiber diameter on the fibrous scaffolds were 250–300 and 15 μm, respectively, by using an X–Y programed collector stage [42]. Melt blowing technology also uses a higher temperature to generate fibrous scaffolds. In melt blowing electrospinning, the polymer is melted and extruded through a jet, while heated air is blown through an air nozzle. Jenkins et al. reported that microarchitectural fibrous scaffolds were produced using a melted polymer with controlled airflow velocities (500–1400 m^3^ air/h/m scaffolds) for rotator cuff tendon tissue engineering [50]. They showed that these fibrous scaffolds have a fiber diameter of 3–5000 nm, which can provide a relevant physiological structure for tendon-like ECM environments.

#### 2.1.3. Micropatterned Collector-Based Electrospinning

Fibrous scaffolds generated using electrospinning are deposited on a conductive metallic collector in a randomly oriented fibrous direction due to their chaotic whipping nature in the electrified liquid jet. These 2D features may be problematic to the broad application of such scaffolds for tissue engineering applications. Zhang et al. utilised a unique patterned collector and fabricated different microscale architectures and macroscale 3D tubular structures [51]. The resulting fibrous scaffolds had hierarchically patterned structures similar to those of the used patterned metallic collector. Another study demonstrated the use of an array of stainless steel beads as the collector, and yielded hierarchically patterned nanoscale fibrous scaffolds with arrayed microscale wells [52]. However, the patterned collectors, which were modulated by weaving or engraving, caused some shortcomings in microscale precision patterning and intricate patterns. In this regard, Liu et al. showed the potential use of a glass template collector patterned with an electrically conductive circuit [43]. They designed a micropatterned glass template on a collector prepared by lithography in order to obtain a micropatterned silver circuit for the selective deposition of electrospun fibers. They demonstrated that lithography could provide a flexible collector surface with microscale precision patterns, and that the cells could be modulated to a precise location and into specific shapes using hierarchically patterned fibrous scaffolds. Furthermore, such strategies using lithographic collectors have also been used for the preparation of fibrous scaffolds for tissue regeneration. For example, Lie et al. fabricated fibrous scaffolds using a lithographic collector with various types of fibrous patterns, such as honeycomb, rectangle, and square shaped, and confirmed that co-cultured cells (cardiomyoblasts, cardiac fibroblasts, and endothelial cells) on honeycomb-patterned fibrous scaffolds showed spontaneous beating similar to that of native cardiomyoblasts [53]. These approaches have yielded materials that mimic the in vivo microenvironment of native tissues, such as spatial arrangement and cell–cell communication, in order to elevate the effects of cell function. Another approach is replacing 2D flat collectors with 3D collecting templates in order to obtain 3D fibrous scaffolds. Zhang et al. introduced methods to fabricate 3D macrofibrous scaffolds with controlled architectures using various 3D collecting templates [51]. They confirmed that 3D fibrous scaffolds with different macroscopic configurations, e.g., length, diameter, and shape, could be fabricated by designing 3D collecting templates.

#### 2.1.4. Post-Processing after Electrospinning

For many years, researchers have attempted to fabricate scaffolds with a hierarchical fibrous structure by generating a nanoscale/microscale structure on prefabricated fibrous scaffolds using chemical modification methods, such as ultraviolet (UV) irradiation [54,55]. However, such conventional fabrication methods for the formation of hierarchical fibrous structures require complicated processes, which tend to result in the inevitable collapse of the fibrous structure and changes in their mechanical properties. To overcome this hurdle, laser processing has been utilised. Laser ablation is a well-known precise fabrication technique that induces negligible thermal stress or collateral damage on the target materials owing to the very short time scales involved in the laser/material interaction (10^−12^ and 10^−15^ s for picosecond and femtosecond lasers, respectively) [56,57]. The laser-based processing of fibrous scaffolds offers several advantages, such as a delicate patterning technique through a rapid and uncomplicated process of direct ablation of the material. Kong et al. used a picosecond laser on aligned nanofibrous scaffolds to create microscale holes (100–200 μm) with a spacing of 50 μm and 200 μm between patterns in engineered corneal tissues [58]. In addition, femtosecond laser patterning can be used to fabricate various patterns on fibrous scaffolds, such as line, well, square, triangle, and pentagon shapes [59,60]. Recent studies have shown that femtosecond laser ablation on fibrous scaffolds may provide an effective in vitro fibrous platform to modulate cell behaviors. Lee et al. employed a femtosecond laser ablation system to create microscale porosity on electrospun nanofibrous scaffolds with diameters of 50 μm, 100 μm, and 200 μm, and spacing of 50 μm and 200 μm between patterns [59]. They demonstrated that femtosecond laser-ablated electrospun fibrous scaffolds not only affected adhesive cell morphology in vitro, but also enabled better cell ingrowth in vivo. Moreover, Jun et al. showed that the orientation of myoblasts could be efficiently modulated by femtosecond laser-ablated microscale grooves on the surface of fibrous scaffolds [44]. They demonstrated that cells initially grew according to the nanoscale random fibrous structure, but eventually reorganised to match the adhesive cellular morphology and orientation of myotube assembly on femtosecond-laser ablated microscale grooves. More importantly, cells showed better ingrowth in laser-ablated fibrous scaffolds than in untreated fibrous scaffolds, which are major drawbacks for conventional electrospinning. Shin et al. evaluated these femtosecond laser-ablated fibrous scaffolds with nanoscale/microscale structures, which were developed to promote the biological function of endothelial cells by resembling the native endothelium [61].

Another simple way to create additional patterns on electrospun fibers is nanoimprinting lithography (NIL). Nandakumar et al. utilised nanoimprinting lithography on electrospun fibers at physiological temperatures [45]. They prepared microscale electrospun fibers (single fibers with a diameter of approximately 6 μm), and then imprinted the fibers within 1–5 min under a high-pressure vacuum at less than 42 °C. Patterns ranging from line to geometric shapes (circles and triangles) were imprinted on prepared fibrous scaffolds.

### 2.2. Three-Dimensional Fibrous Scaffolds

**Figure 3:**
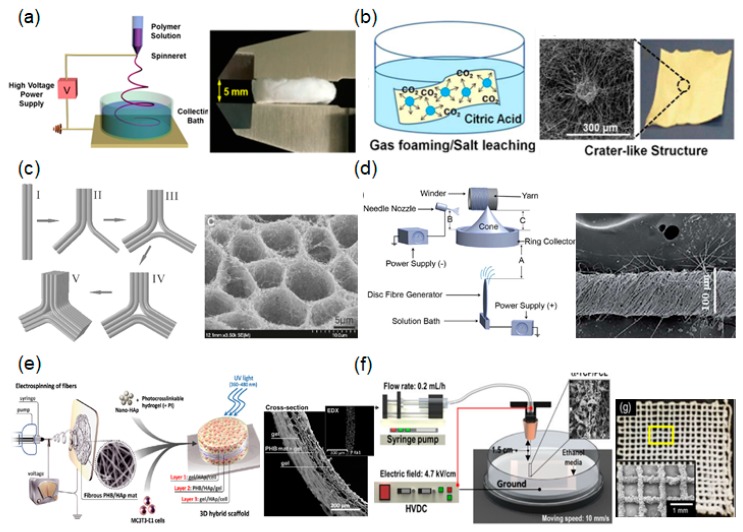
Several approaches to the formation of three-dimensional (3D) fibrous scaffolds using different electrospinning process: (**a**) liquid-collecting electrospinning [62]; (**b**) gas foaming [63]; (**c**) self-assembly [64]; and (**d**) fibrous yarn scaffolds [65]; (**e**) schematic illustration of a hydrogel-integrated fibrous scaffold [66]; (**f**) a hybrid system using 3D printing and electrospinning [67].

The fibrous scaffolds produced by conventional electrospinning are usually two-dimensional rather than three-dimensional, hampering cell infiltration. Although several approaches have been explored to increase the porosity of electrospun fibrous scaffolds to overcome cell ingrowth, these approaches do not produce 3D scaffolds with respect to thickness. Several approaches to form 3D fibrous scaffolds using different electrospinning processes have been developed (Figure 3). 

#### 2.2.1. Liquid-Collecting Electrospinning

A technique using liquid reservoirs as collectors has attracted attention as a method for preparing 3D fibrous scaffolds. The utilization of liquids with low surface tension, such as water, ethanol, and methanol, causes the extruded fibers to sink during electrospinning, and overcomes the effects of fiber bonding. This results in the loosening of fibrous layers of scaffolds with higher internal porosity. The choice of the liquid to use in the reservoir should be considered according to surface tension and hydrophilicity. For example, Chen et al. fabricated 3D fibrous scaffolds incorporating fibrous morphologies and interconnected pore structures using liquid-collecting electrospinning. In their study, a bath containing diluted alcohol solution was used as the liquid-based collector [62]. During this process, electrospun fibers continuously accumulated in relatively fluffy stacks. After the removal of alcohol through a freezing process at a low temperature, foamed 3D fibrous scaffolds were prepared. The porosity of the formed 3D fibrous structures was much larger than that of conventional fabricated 2D electrospun fibrous scaffolds. The thickness of the fibrous scaffolds prepared by this method was measured as 5 mm. Recently, Kasuga et al. successfully fabricated macroscale 3D fibrous scaffolds (with a thickness of 40–50 mm) with a cotton-like structure using this method [68]. They claimed that these 3D fibrous scaffolds could improve the localization of neighboring cells at the initial stage after implantation.

#### 2.2.2. Gas Foaming

Three-dimensional fibrous scaffolds using gas foaming provide viable alternatives to open the pores between fibrous networks with a minimal application of force. The gas foaming technique for prepared 2D fibrous scaffolds usually involves three steps: 2D fibrous scaffold/gas solution formation, gas bubble nucleation/growth, and 2D fibrous scaffold expansion to 3D fibrous scaffolds. Jiang et al. reported on the feasibility of this process using prepared 2D fibrous scaffolds and NaBH_4_ as a gas foaming agent [69]. The prepared 2D fibrous scaffolds were soaked in NaBH_4_ solution, and bubbles were then formed/generated on the 2D fibrous scaffolds, creating a more open scaffold. Researchers demonstrated that a higher concentration of gas foaming agent significantly increased the thickness of the prepared 2D fibrous scaffolds. In addition, cells successfully infiltrated and grew throughout the gas foamed 3D fibrous scaffolds. A similar technique was reported by Hwang et al. involving the use of a gas foaming/salt leaching technique [63]. Briefly, Hwang et al. fabricated fibrous scaffolds with crater-like structures, enabling them to replicate the 3D ECM fibrous environment. Notably, this scaffold permitted human mesenchymal stem cells to penetrate through the prepared 3D fibrous scaffold (up to 250 μm), whereas most human mesenchymal stem cells were not able to penetrate through the conventional electrospun fibrous scaffolds within seven days.

#### 2.2.3. Self-Assembly

Self-assembly is a process in which a disordered arrangement forms an ordered system as a consequence of specific, local interactions among the components themselves. Self-assembly of the fibrous scaffolds was investigated for several types of polymers, including polyethylene oxide (PEO), polyacrylonitrile (PAN), PCL, and polyvinyl alcohol. Liang et al. utilised precursor polymer solutions (PEO dissolved in water) and fabricated self-assembled fibrous structures with honeycomb patterns [70]. They explained the relationship between the concentration of the polymer solution and the self-assembled formation of honeycomb patterns. In addition, Ahirwal et al. fabricated self-organised fibrous structures in honeycomb patterns using a prepared polymer solution (PCL dissolved in dimethylformamide (DMF)) [71]. Yan et al. studied the mechanisms underlying this phenomenon, and found that the polymer solution concentration, collected substrates, collection distance, and humidity played critical roles in the formation of the self-assembled honeycomb-patterned structures in fibrous scaffolds [64]. For example, self-assembled honeycomb patterns were not observed at higher concentration (i.e., 7% PAN dissolved in DMF). The well-defined 3D honeycomb structures in fibrous scaffolds could only form when the humidity decreased below 60% for the PAN/DMF solution. Moreover, the self-assembled pore size increased as the distance between the polymer jet and collector decreased.

#### 2.2.4. Fibrous Yarn Scaffolds

Based on the hierarchical architecture of soft tissues, such as tendon and ligaments, the fabrication of multifilament yarn scaffolds has been highlighted for tissue engineering applications [72]. Yarns are linear assemblies of single fibers with improved mechanical properties generated through twisting, weaving, and knitting. Recently, considerable efforts have been made to fabricate fibrous yarn scaffolds directly through electrospinning. For example, Ali et al. fabricated continuous and twisted fibrous yarns using a multi-nozzle and rotary funnel collector [65]. They demonstrated that the flow rate of the polymer solution and the speed of the rotating collector could control fibrous yarn production and twist rates. Additionally, Mouthuy et al. used a textile production line to create multifilament yarn, which could mimic the hierarchical architecture of the tendon [73]. Fibrous yarn scaffolds prepared using this method improved the functions of primary human tenocytes, including adherent cell numbers and proliferation. Furthermore, they directly implanted fibrous yarn scaffolds into transected infraspinatus tendons, and confirmed the good safety profile of this method, with only a mild foreign body reaction. Chang et al. reported on twisted fibrous yarns consisting of microfiber and nanofiber yarns that were prepared using a high-speed spinneret tip [74]. With the high rotational speed of the spinneret, charged fibers could be deposited on the ground collector with rotation during jetting to form a twisted fibrous rope. The formation of twisted fibrous yarn is dependent on the distance between the spinneret tip and the ground collector, and such fibrous yarn scaffold systems can be utilised not only for enhancing mechanical strength, but also for generating medical scaffolds with improved therapeutic effects.

#### 2.2.5. Hydrogel-Integrated Fibrous Scaffolds

Hydrogel-integrated fibrous scaffolds may also be used to mimic the native tissue environment. An advantage of combining fibrous scaffolds with hydrogels is that cells can easily migrate through 3D environments, such as the 3D environment of native ECM. Indeed, hydrogel-integrated fibrous scaffolds allow for cellular contact guidance within 3D environments, which cannot be accomplished with separate hydrogel or electrospun fiber systems. Sadat-Shojai et al. attempted to reconstruct the structure using electrospun fibers as the inner layer, and a hydrogel as the 3D structure [66]. Briefly, they prepared an electrospun fibrous layer that was soaked in the precursor gel mixture with a photoinitiator. The 3D hydrogel integrated fibrous scaffold was formed by UV light for 10 s. The inner electrospun fibers were designed to provide mechanical support, while the embedded hydrogels were designed to facilitate cell proliferation and spread. Similarly, Wu et al. prepared 3D hybrid scaffolds based on a fibrous yarn network within a hydrogel shell to mimic the native cardiac tissue structure [75]. They prepared an interwoven aligned fibrous structure via a weaving technique, and then encapsulated the structure within photocurable hydrogels. These 3D hybrid scaffolds promoted the alignment of cardiomyoblasts on each fibrous layer, and individually controlled the cellular orientation of different layers in a 3D hydrogel environment.

#### 2.2.6. Near-Field Electrospinning (NFE) with 3D Printing Technology

Despite extensive studies, it is still not possible to fabricate highly organised fibrous scaffolds with controlled uniformity and architecture using conventional electrospinning generated by the chaotic whipping of liquid jets. NFE is a relatively new method in the field of electrospinning, and has recently been actively applied by researchers. NFE uses a short distance between the spinneret and collector (less than 3 mm) to prevent the bending instability and split in ejected fibers. This method stabilizes the region of the ejected polymer jet to control fiber deposition and enable the production of 3D structures. For example, Lu et al. introduced an NFE method to fabricate 3D microstructures of fibrous scaffolds [76] by balancing the distance between the spinneret and the collector (0.5–3 mm). In addition, a programmable X–Y motion stage was also utilised to deposit the fibrous structures in a predesigned path.

Alternatively, an electrohydrodynamic printing process was developed to deposit the electrohydrodynamically printed fibers into customised patterns by controlling the X–Y motion stage [67]. Briefly, this system enabled microscale fibrous bundles to form from the charged single jet by replacing the solvent of the polymeric solution with an alcohol-based solvent. They determined the optimal processing conditions, including electric field, distance, flow rate, and needle gauge, in order to fabricate 3D microscale fibrous scaffolds. Fibrous scaffolds prepared using this technique promoted osteoblast behaviors and functions.

Fattahi et al. designed 3D fibrous scaffolds in combination with 3D printing technology and NFE [77]. This approach yielded highly organised fibrous architectures with the desired form via an X–Y–Z moving stage. Additionally, He et al. combined melt electrospinning with 3D printing. The ejected melted fibers could be precisely stacked in a layer-by-layer manner to form 3D fibrous scaffolds [78]. Three-dimensional printing and electrospinning are relatively new in the field of electrospinning and have recently been actively applied by researchers. A hybrid system using 3D printing and electrospinning can help create 3D fibrous scaffolds with similar complex architectures and the ability to regulate cellular behaviors.

## 3. Conclusions and Future Perspectives

The native tissue has complex structures with somewhat unique arrangements and architectures of fibrous shapes, and ECM-mimicking materials have been of great interest to scientists, particularly in the field of tissue engineering. Electrospinning offers advantages for the preparation of fibrous scaffolds resembling the fibrillar architecture of the ECM in native tissues, yielding materials with major advantages in tissue engineering. There are many methods for fabricating 2D and 3D fibrous scaffolds, which show structural characteristics similar to those of the native ECM. In this review, we discussed recent advances in the fabrication of fibrous scaffolds with desired geometries and architectures using several electrospinning techniques. This review provided an overview of the fundamental principles of the electrospinning process for generating complex fibrous scaffold geometries similar to the structural complexity of the ECM in living tissue. As our understanding of the origins of these features increases, we can begin to design, fabricate, and apply this knowledge in the biomedical field.

Although there are still many challenges to overcome, electrospinning shows enormous potential in the fabrication of fibrous scaffolds with controllable geometric/architectural structures, enabling researchers to design and develop novel fibrous scaffolds that more closely mimic the structural environment of the native ECM. In the development of “ECM-mimicking materials,” the objective is to improve our understanding of native complex structures and prepare highly efficient biomedical scaffolds for tissue engineering. Further studies of fibrous scaffolds are ongoing, and will be useful for achieving efficient tissue engineering.
